# Coordination and Variation of Photosynthetic Pigments Across Seedling Ages and Grades Reveal Adaptive Strategies in *Pinus yunnanensis*

**DOI:** 10.3390/biology15201844

**Published:** 2026-10-14

**Authors:** Ye Jiang, Zhuangyue Lu, Qingxue Zhang, Tianyi Xue, Xiangyang Kang, Yulan Xu, Nianhui Cai, Lin Chen

**Affiliations:** 1 The Key Laboratory of Forest Resources Conservation and Utilization in the Southwest Mountains of China, Ministry of Education, Southwest Forestry University, Kunming 650233, China; 2 Key Laboratory of National Forestry and Grassland Administration on Biodiversity Conservation in Southwest China, Kunming 650224, China; 3 State Key Laboratory of Tree Genetics and Breeding, National Engineering Research Center of Tree Breeding and Ecological Restoration, College of Biological Sciences and Biotechnology, Beijing Forestry University, Beijing 100083, China

**Keywords:** seedling grading, photosynthetic pigments, allometric growth, coefficient of variation, seedling-quality evaluation

## Abstract

*Pinus yunnanensis* is a vital native species for afforestation in Southwest China, and seedling quality determines restoration outcomes. Seedling grading is widely applied to select robust seedlings, whereas physiological distinctions among seedlings of different grades remain unclear. In the present study, allometric scaling, coefficient-of-variation calculation and correlation analysis are adopted to investigate pigment traits and resource-allocation characteristics of Grade I, Grade II and Grade III seedlings across successive developmental stages. Seedling grade and seedling age exert significant influences on photosynthetic pigment traits. Grade I seedlings exhibit stable pigment composition, steady allometric growth and strong phenotypic stability, with key physiological transitions taking place at 330 d and 510 d. Chlorophyll b showed the highest trait variability. The present results verify the superior adaptability of Grade I seedlings and provide theoretical support for early seedling-quality evaluation and precise seedling cultivation.

## Simple Summary

*Pinus yunnanensis* is a key native tree species for forest restoration in Southwest China. High-quality seedlings are critical for successful afforestation. Forest practitioners classify seedlings into different grades, yet physiological differences among these graded seedlings remain poorly understood. Leaf-pigment and growth patterns of graded *Pinus yunnanensis* seedlings across successive growth stages are analyzed in this study. Grade I seedlings maintain a more stable physiological status and stronger adaptability, with obvious physiological shifts occurring at two important developmental stages. Chlorophyll b represents the most variable leaf pigment. This work reveals the mechanisms underlying the superior performance of Grade I seedlings. The findings support early-stage judgment of seedling quality and provide guidance for scientific seedling cultivation for ecological afforestation.

## 1. Introduction

*Pinus yunnanensis* Franch. is a tree species endemic to the southwestern China. This species is primarily distributed in western areas at elevations ranging from 1500 to 3000 m [1]. As a major economic and afforestation tree species, it plays an indispensable role in woodland construction and has substantial developmental potential. The early-stage biomass development of *P. yunnanensis* seedlings significantly impacts their future survival rates. Evaluating the early growth performance of forest trees can provide a basis for subsequent seedling cultivation and reduce the time required for forestation [2]. Therefore, investigating the quality grading of *P. yunnanensis* seedlings is essential. Internationally, practical seedling-grading systems still rely heavily on morphological measurements, while physiological indicators such as photosynthetic pigments remain under-exploited for routine nursery quality assessment [3,4]. In Europe, different countries have established national standards and evaluation frameworks for forest reproductive materials [5]. For example, Germany, France and Spain have developed distinct testing systems for seed provenance, seedling morphological and physiological traits, aiming to guarantee the quality of planting stocks used in afforestation. Although these European assessment systems mainly focus on temperate tree species, their combined use of morphological indicators and physiological biomarkers provides valuable references for evaluating seedling quality of native conifers in Southwest China. The mean ± standard deviation method, which is more consistent with production practice, is adopted. Previous studies have indicated that the classification outcomes for seedlings can vary under different grading standards; therefore, multiple indicators are typically used as criteria for evaluating and classifying seedling grades [6]. Photosynthetic-related physiological traits of conifer seedlings are sensitive to external conditions, which can be reflected by shifts in pigment performance [7]. Photosynthetic pigment content serves as a critical indicator reflecting the photosynthetic physiological status of *P. yunnanensis*, sensitively signaling its growth vitality and responses to environmental stresses such as drought. By analyzing differences in photosynthetic pigment content among seedlings of different grades, it is possible to determine whether high-quality vigorous seedlings possess inherently higher light utilization efficiency physiologically. This facilitates the integration of photosynthetic pigment parameters into the seedling-quality evaluation system, offering novel reference indicators for the early and rapid screening of provenances or families with growth advantages. *P. yunnanensis* exhibits flexible growth and resource-allocation strategies, resulting in distinct biomass accumulation and allocation patterns in seedlings across different seasons [8]. In plants, biomass accumulation and allocation represent not only the net outcome of carbon gain but also key drivers of plant growth [9]. Photosynthetic pigments constitute the physiological foundation for biomass accumulation during photosynthesis, and photosynthesis is closely linked to chlorophyll content [10]. Photosynthetic performance of conifer seedlings changes across ontogenetic stages and seasonal time courses, which shapes their long-term growth performance [11]. The content of chlorophyll (total chlorophyll, chlorophyll a, chlorophyll b) and accessory pigments such as carotenoids in leaves not only reflects the photosynthetic capacity and resource acquisition efficiency of plants but is also commonly used as a crucial indicator of plant growth condition [12]. Consequently, investigating *P. yunnanensis* seedlings from the perspective of photosynthetic pigments, which possess higher sensitivity to environmental changes, allows for a more intuitive analysis of seedling growth patterns. Numerous contemporary studies have employed this methodology to investigate allometric growth in plants [13,14].

Allometric growth refers to the disproportionate scaling relationship between two traits in an organism [15], reveal the intrinsic rules governing plant growth [16,17,18]. Through allometric analysis, researchers can gain deeper insights into plant phenotypic plasticity and understand plant adaptation mechanisms. From an allometric perspective, plant resource allocation is an individual size-dependent process; that is, allometry quantitatively describes the relationship between plant growth and resource allocation [19,20]. Considerable intraspecific variation exists among conifer seedlings, and such phenotypic differences are closely linked to seedling stress adaptability for forest restoration purposes [21]. Nevertheless, interpretations of these among-trait CV differences require caution, as direct cross-trait comparison of coefficient of variation may be biased by trait-specific scaling properties [22]. Furthermore, allometric analysis unveils the intrinsic interconnections within plant growth, which is not only conducive to in-depth theoretical research in botany but also holds significant implications for practical applications in production [23]. Nevertheless, few studies have integrated photosynthetic pigment dynamics with seedling-grade classification for native subtropical conifers across successive ontogenetic stages [24]. Accordingly, this study investigated photosynthetic pigment dynamics, allometric growth patterns and trait variation coefficients among *Pinus yunnanensis* seedlings of different grades across multiple developmental stages, aiming to clarify how seedling grade shapes physiological adaptation and resource-allocation strategies, and to provide physiological reference for seedling-quality evaluation of this native conifer species.

## 2. Materials and Methods

### 2.1. Study Site Description

The experiment was conducted in a greenhouse at Southwest Forestry University, Kunming City, Yunnan Province (25°04′00″ N, 102°45′41″ E) at an altitude of approximately 1945 m. The soil is classified as acidic, phosphorus-deficient red soil [25].

### 2.2. Experimental Materials

#### 2.2.1. Seed Source

The experimental seeds were collected from a clonal seed orchard of *Pinus yunnanensis* in Midu County. The mother trees were in good growing condition, and plump, mature cones of the current year were collected from the improved variety base of *P. yunnanensis*. Bulked mixed seeds from multiple clones within the seed orchard were used. Seeds were fully blended prior to sowing, and the exact number of sampled mother trees was not recorded for this experiment. The cones were labeled, transported to the laboratory, and sun-dried. Seeds that were plump and uniform in size were selected. The selected seeds were disinfected with 0.5% KMnO_4_ solution for 1 h, rinsed with clean water, and then soaked in warm water for 24 h. Similarly, the soil and seedling trays were disinfected with KMnO_4_ solution. Sowing was conducted at the end of January in the first year. Subsequently, the seedbeds were covered with pine needles, and a shed was set up and covered with plastic film. Routine management practices, including regular watering and weeding, were carried out. At the end of the seed germination phase, in May of the first year, the 100 d seedlings were transplanted into nursery pots (specifications: bottom diameter 18 cm × height 32 cm). The substrate consisted of humus soil and red soil mixed in a ratio of 3:1. Seedlings with uniform individual size were selected for subsequent experiments. During the experimental period, seedlings were managed uniformly, with weeding every two weeks and watering every 3–5 d, ensuring thorough watering each time.

#### 2.2.2. Seedling Grading

All *P. yunnanensis* seedlings were cultivated under identical environmental conditions in a completely randomized common-garden experiment. In total, 3017 seedlings were surveyed to calculate seedling-grading thresholds at 240 d after sowing. The mean ± 1/2 σ standard deviation method (*X* ± 1/2 σ) was adopted, where *X* is the mean seedling height and σ is the population standard deviation of seedling height. Descriptive statistics for initial seedling height, grading thresholds and sample sizes for each seedling grade are summarized in Supplementary Table S1.

Based on the above-calculated thresholds, more than 1900 seedlings were selected and classified into three grades (Grade I, Grade II and Grade III) [26]. The grading criteria were as follows:

Grade I: I ≥ *X* + 1/2 σ;

Grade II: X − 1/2 σ ≤ II < *X* + 1/2 σ;

Grade III: III < *X* − 1/2 σ. 

The grade order was Grade I > Grade II > Grade III. After grading, seedlings of different grades were cultivated under the same conditions for subsequent experiments [27].

### 2.3. Measurement of Indicators

#### Determination of Photosynthetic Pigment Content

The samples for photosynthetic pigment quantification were collected at six ontogenetic stages: 240 d, 330 d, 420 d, 510 d, 600 d, and 690 d after sowing. Three independent composite biological samples were prepared for each seedling grade at each sampling date. Each composite sample was pooled with needles from 9 randomly selected seedlings. Three technical replicates were performed on each composite sample for photosynthetic pigment measurement. The extraction procedure and calculation formulas followed those described in a previous study by the research group [28]. The calculation formulas are as follows:

Chlorophyll a content (mg/g) = (12.72A_663_ − 2.59A_645_) × V/1000 m

Chlorophyll b content (mg/g) = (22.88A_645_ − 4.67A_663_) × V/1000 m

Total chlorophyll content (mg/g) = (20.2A_645_ − 8.02A_663_) × V/1000 m

Carotenoid content (mg/g) = (1000A_470_ − 3.27Ca − 104Cb)/229 × V/1000 m

Note: In the formulas, V represents the total volume of the extract (mg); m represents the fresh weight of the sample (g); Ca represents the concentration of chlorophyll a (mg/g); Cb represents the concentration of chlorophyll b (mg/g). A663: absorbance measured at a wavelength of 663 nm; A645: absorbance measured at a wavelength of 645 nm; A470: absorbance measured at a wavelength of 470 nm.

### 2.4. Data Analysis

Data were organized using Microsoft Excel 2020. Descriptive statistical analysis was performed with IBM SPSS Statistics 27. Two-way analysis of variance (ANOVA) was performed to test the main effects of seedling age, seedling grade, and their interaction on photosynthetic pigment contents and pigment ratios. Tukey’s HSD post hoc test was conducted for pairwise multiple comparisons when ANOVA revealed significant effects, to generate significance letters shown in figures. Pearson correlation analysis was employed to evaluate the relationships among different seedling ages, grades, and photosynthetic pigments. The coefficient of variation (CV) was calculated using the formula CV = Standard Deviation/Mean × 100% [1]. The allometric relationship is typically described using the equation: *y* = a*x*^b^. This equation can be linearized as: log *y* = log a + b log *x* [29], where *x* and *y* represent different photosynthetic pigments, including total chlorophyll. In this equation, a is the intercept, representing the baseline trait, and the slope b is the allometric growth index. When b has no significant difference from 1, the relationship is considered isometric; when b is significantly different from 1, it indicates allometric growth [1]. Differences in slopes among groups were compared, and if no significant differences were found, a common slope was reported. *p* < 0.05 indicates that the fitting result is significant, while *p* > 0.05 suggests that the fitting result is not significant. Parameter estimation for the allometric model was conducted using the standardized major axis (SMA) regression method, implemented via the “Smatr” package version 3.4-8 in R language [30,31]. Graphs were generated using Origin 2024. It should be noted that total chlorophyll and multiple pigment ratios are mathematically derived variables sharing identical raw pigment components; such mathematical dependency may partly contribute to observed correlation and allometric relationships, and thus results should be interpreted with caution.

## 3. Results

### 3.1. Effects of Photosynthetic Pigment Content on Height Growth Variation Among P. yunnanensis Seedlings of Different Grades

#### 3.1.1. Chlorophyll a Content in Different Grades of *P. yunnanensis*

**Figure 1:**
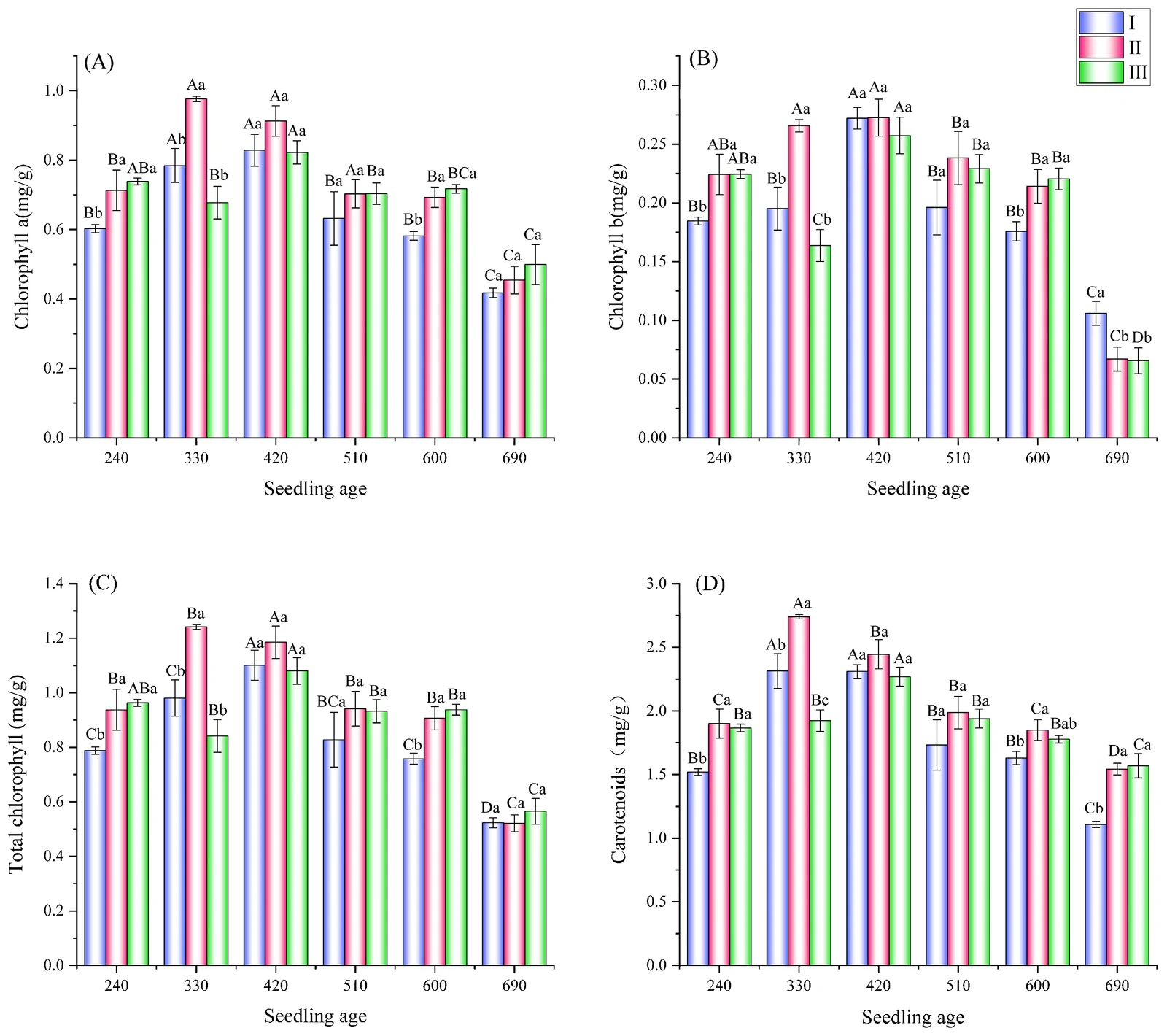
Dynamics of photosynthetic pigment content in different grades of *P. yunnanensis* seedlings over time. Note: Panels (**A**–**D**) represent chlorophyll a, chlorophyll b, total chlorophyll, and carotenoid concentrations, respectively. Bars in blue, red and green correspond to Grade I, Grade II and Grade III seedlings. Data are presented as mean ± standard error. Capital letters denote significant differences among seedling ages within the same seedling grade; lowercase letters denote significant differences among seedling grades at the same seedling age (*p* < 0.05).

Significant differences in chlorophyll a content were observed among different grades across various ages over time (*p* < 0.05, similarly hereafter), indicating that seedling grade and age significantly influenced chlorophyll a content (Figure 1A). Among these, Grade II exhibited the maximum chlorophyll a content at 330 d, whereas Grade I and III reached their maximum at 420 d. At 240 d, 600 d, and 690 d, chlorophyll a content in Grade II and III was significantly higher than in Grade I. At 330 d, Grade II showed significantly higher chlorophyll a content compared to Grade I and III. At 420 and 510 d, no significant differences in chlorophyll a content were detected among the different grades. Meanwhile, Grade I and II displayed similar temporal trends, characterized by an initial increase followed by a decrease.

#### 3.1.2. Chlorophyll b Content in Different Grades of *P. yunnanensis*

Significant differences in chlorophyll b content were found among different grades across various ages over time, with seedling grade and seedling age exerting significant effects on chlorophyll b content (Figure 1B). Grade II attained their maximum chlorophyll b content at 330 and 420 d, while Grade I and III peaked at 420 d. At 240 and 600 d, chlorophyll b content in Grade II and III was significantly higher than in Grade I. At 330 d, Grade II exhibited significantly higher chlorophyll b content compared to Grade I and III. At 420 and 510 d, no significant differences in chlorophyll b content were observed among seedling grades. At 690 d, Grade I showed significantly higher chlorophyll b content than Grade II. Furthermore, Grade I and II demonstrated similar temporal patterns, although Grade I exhibited greater amplitude of variation compared to Grade II.

#### 3.1.3. Carotenoids Content in Different Grades of *P. yunnanensis*

Significant differences in carotenoids content were detected among different grades across various ages over time, with seedling grade and age significantly affecting carotenoids content (Figure 1C). Grade I reached their maximum carotenoids content at 330 and 420 d, Grade II peaked at 330 d, while Grade III attained their maximum at 420 d. At 240 d, carotenoids content in Grade II and III was significantly higher than in Grade I. At 330 d, carotenoids content followed the order: Grade II > Grade I > Grade III. At 420 and 510 d, no significant differences in carotenoids content were found among the three grades. At 600 d, Grade II exhibited significantly higher carotenoids content than Grade I. At 690 d, carotenoids content in Grade II and III was significantly higher than in Grade I. Additionally, Grade I and II displayed an initial increase followed by a decrease over time, with Grade II consistently maintaining higher carotenoids content than Grade I and III throughout the observation period.

#### 3.1.4. Total Chlorophyll Content in Different Grades of *P. yunnanensis*

Significant differences in total chlorophyll content were observed among different grades across various ages over time, indicating that seedling grade and age significantly influenced total chlorophyll content (Figure 1D). Grade II exhibited maximum total chlorophyll content at 330 d, whereas Grade I and III reached their maximum at 420 d. At 240 and 600 d, total chlorophyll content in Grade II and Grade III was significantly higher than in Grade I. At 330 d, Grade II showed significantly higher total chlorophyll content compared to Grade I and III. At 420, 510 and 690 d, no significant differences in total chlorophyll content were detected among the different grades. Furthermore, Grade I and II demonstrated similar temporal patterns characterized by an initial increase followed by a decrease; however, Grade I consistently exhibited lower total chlorophyll content than Grade II throughout the study period.

### 3.2. Dynamic Changes in Photosynthetic Pigment Ratios of P. yunnanensis Seedlings at Different Ages and Grades

**Figure 2:**
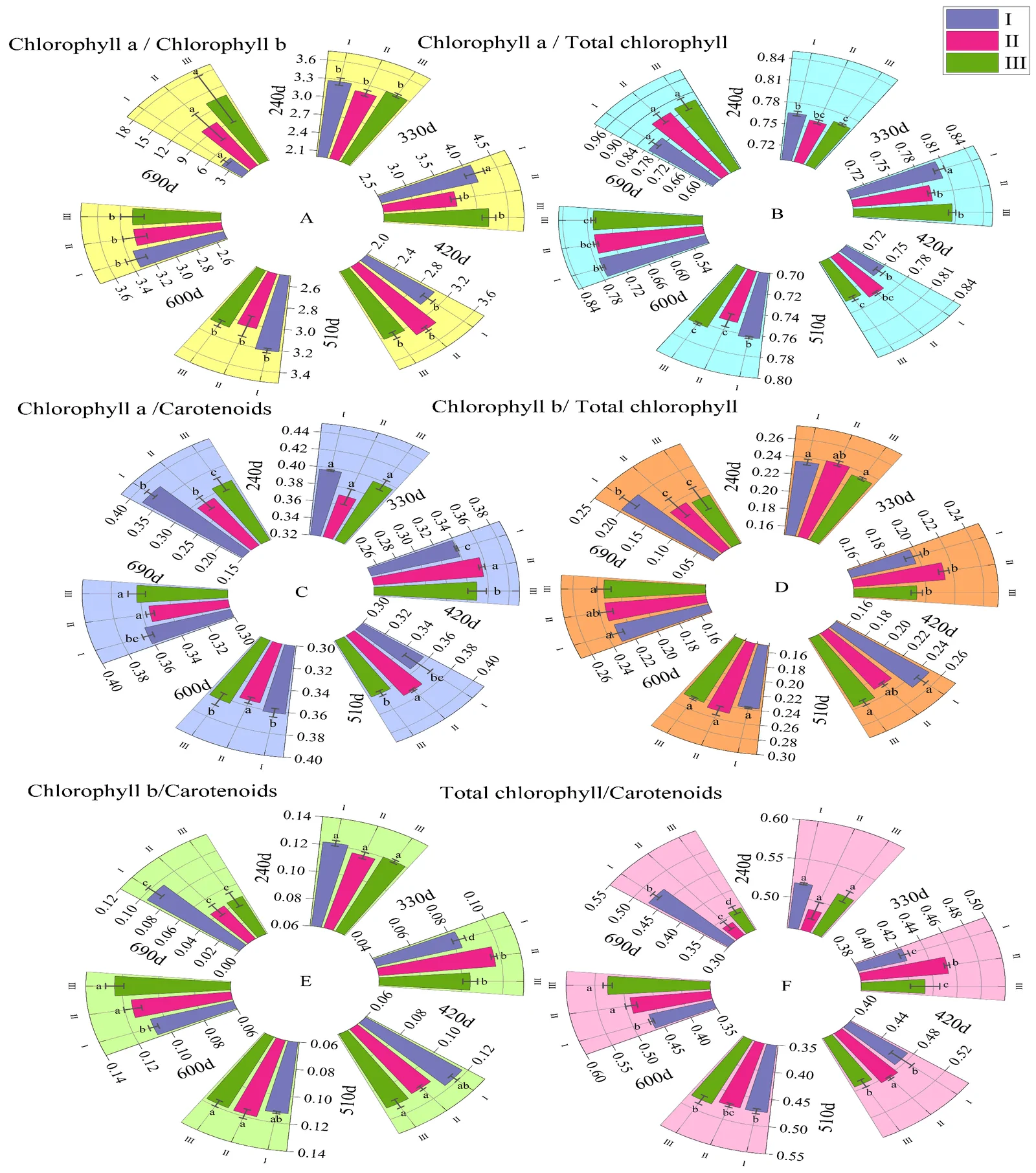
Pie chart of the ratios of different photosynthetic pigments to carotenoids at different seedling ages. Note: Panels (**A**–**F**) represent chlorophyll a/chlorophyll b, chlorophyll a/total chlorophyll, chlorophyll a/carotenoids, chlorophyll b/total chlorophyll, chlorophyll b/carotenoids, and total chlorophyll/carotenoids, respectively. Colored sectors in each polar plot correspond to six seedling ages (240 d, 330 d, 420 d, 510 d, 600 d, 690 d). Blue, red and green indicate Grade I, Grade II and Grade III seedlings. Different lowercase letters indicate significant differences (*p* < 0.05) among seedling ages within the same seedling grade.

The chlorophyll a/chlorophyll b ratio (Figure 2A) was significantly higher in Grade I than in Grade II and III at 240 d, 330 d, 600 d and 690 d (*p* < 0.05). Notably, the ratio in Grade I peaked at the mature seedling stage (690 d), reaching 1.67 times that of Grade II. The chlorophyll a/total chlorophyll ratio exhibited dynamic changes with seedling age (Figure 2B): the ratio was highest at 240 d, indicating high activity of the photosynthetic reaction center and rapid growth; it then decreased continuously from 330 d to 510 d, accompanied by an increased proportion of chlorophyll b.

The chlorophyll a/carotenoids ratio of *P. yunnanensis* seedlings varied significantly with both seedling age and grade (Figure 2C). Across all grades, the chlorophyll a/carotenoids ratio showed a unimodal trend with increasing age, peaking at 330 d and reaching its minimum at 690 d. For Grade I, the chlorophyll a/carotenoids ratio was significantly higher than that of Grade II and III at most ages (240 d, 420 d, 600 d, 690 d), indicating superior photosynthetic activity. Grade II showed a mid-term growth advantage, with the highest chlorophyll a/carotenoids ratio at 510 d, which was significantly higher than that of Grade I and III. Grade III maintained the lowest chlorophyll a/carotenoids ratio at most ages, suggesting a higher proportion of carotenoids and stronger photoprotective requirements.

The chlorophyll b/total chlorophyll ratio showed a bimodal trend with increasing seedling age (Figure 2D), with the first peak at 240 d, the second peak at 600 d, and the lowest value at 690 d. For Grade I, the chlorophyll b/total chlorophyll ratio remained at a relatively high level at most ages (240 d, 420 d, 600 d), indicating stronger low-light adaptation capacity. The ratio in Grade III was comparable to that of Grade I at 240 d, but significantly higher than that of Grade I at 690 d, reflecting enhanced low-light harvesting ability by increasing the light-harvesting antenna size. As previously described, from 600 d to 690 d, the chlorophyll a/total chlorophyll ratio recovered, with the proportion of chlorophyll a increasing, indicating maturation of the photosynthetic system. Grade I consistently maintained a higher chlorophyll a/total chlorophyll ratio throughout the entire growth period, showing a significant advantage in photosynthetic efficiency; Grade III had the lowest ratio at most ages, indicating the weakest photosynthetic capacity; Grade II were intermediate between the two, with no significant difference from Grade I at the mature stage (690 d).

The chlorophyll b/carotenoids ratio of *P. yunnanensis* seedlings exhibited significant seasonal fluctuations, with divergent adaptive strategies among different seedling grades (Figure 2E). Upon entering winter (330 d, 690 d), the ratio decreased sharply in all grades, with Grade I high-quality seedlings showing significantly lower values than Grade II and III.

The total chlorophyll/carotenoids ratio (Figure 2F) reached its absolute peak at 600 d, a secondary peak at 240 d, and the lowest value at 690 d. For Grade I, the total chlorophyll/carotenoids ratio was significantly higher than that of Grade II and III at 240 d, 330 d, and 690 d. Grade II showed an obvious mid-term growth advantage, with the highest total chlorophyll/carotenoids ratio at 420 d and 510 d, which was significantly higher than that of Grade I and III.

### 3.3. Variation Characteristics of Photosynthetic Pigments Under Different Seedling Ages and Grades

**Figure 3:**
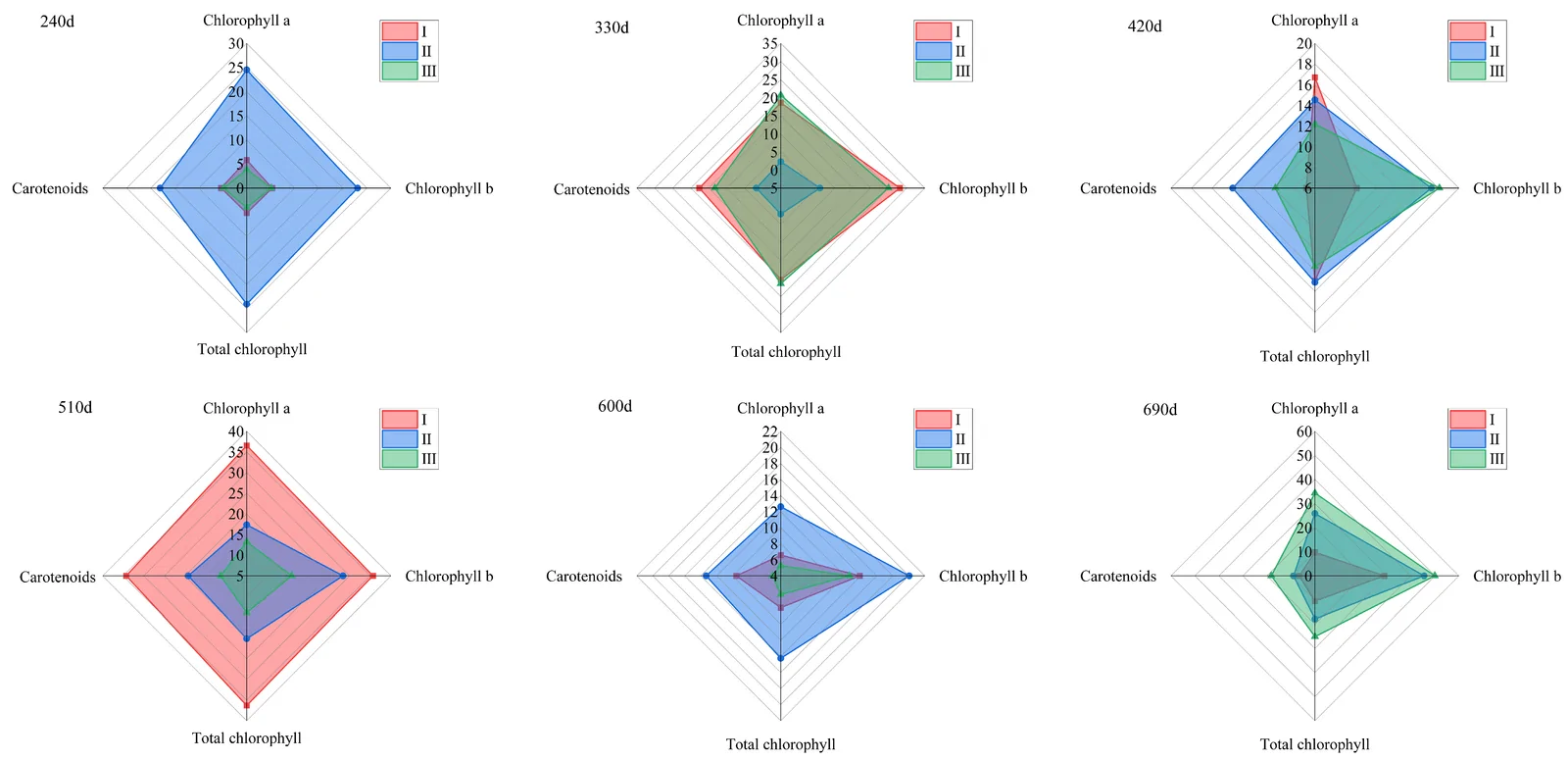
Coefficients of variation (CV) of photosynthetic pigment contents in *P. yunnanensis* seedlings at different seedling age levels. Note: Each subplot corresponds to one seedling age. Axes of radar plots represent chlorophyll a, chlorophyll b, total chlorophyll, and carotenoids, respectively. The extension of the polygon indicates the magnitude of the coefficient of variation (CV); larger polygon area corresponds to higher CV value. Different-colored polygons denote three seedling grades (Grade I, Grade II, Grade III).

The overall coefficients of variation (CV) for each pigment ranked as follows: chlorophyll b > chlorophyll a > total chlorophyll > carotenoids (Figure 3). Chlorophyll is a key pigment involved in photosynthesis, and its content directly affects photosynthetic efficiency. At 240 d and 330 d (Figure 3), the CV values of photosynthetic pigments in seedlings of all grades were generally high, particularly in Grade I, indicating pronounced differentiation in photosynthetic pigments during the early stage and intense competition. At 420 d and 510 d (Figure 3), the CV of Grade I began to decline, while Grades II and III still exhibited relatively high CV values, suggesting that Grade I tend to stabilize in terms of photosynthetic pigments. By 690 d (Figure 3), the CV of Grade I significantly decreased for all pigments, whereas Grades II and III still showed some variation. Overall, Grade I had the lowest CV among all seedling ages, especially for chlorophyll a and total chlorophyll, indicating a more stable photosynthetic pigment composition and stronger adaptability. Grade II generally exhibited the highest CV, particularly during the early–mid and mid stages, reflecting greater individual differentiation. Meanwhile, the CV values of chlorophyll a and total chlorophyll were relatively high across all grades and seedling ages. Across the full dataset covering all ages and seedling grades, chlorophyll b exhibited the highest overall coefficient of variation among the four photosynthetic pigments. Nevertheless, at some individual sampling time points, chlorophyll b could show relatively low CV values for certain seedling grades. As an accessory light-harvesting pigment, chlorophyll b was more sensitive to individual seedling differences, seedling grade and environmental fluctuation, showing pronounced phenotypic variation through ontogenetic development. Carotenoids maintained comparatively lower overall variation across most conditions. Elevated CV values across all seedling grades were observed at 510 d.

### 3.4. Allometric Growth Relationships Between Chlorophyll a and Chlorophyll b Contents in P. yunnanensis Seedlings at Different Seedling Age Grades

**Figure 4:**
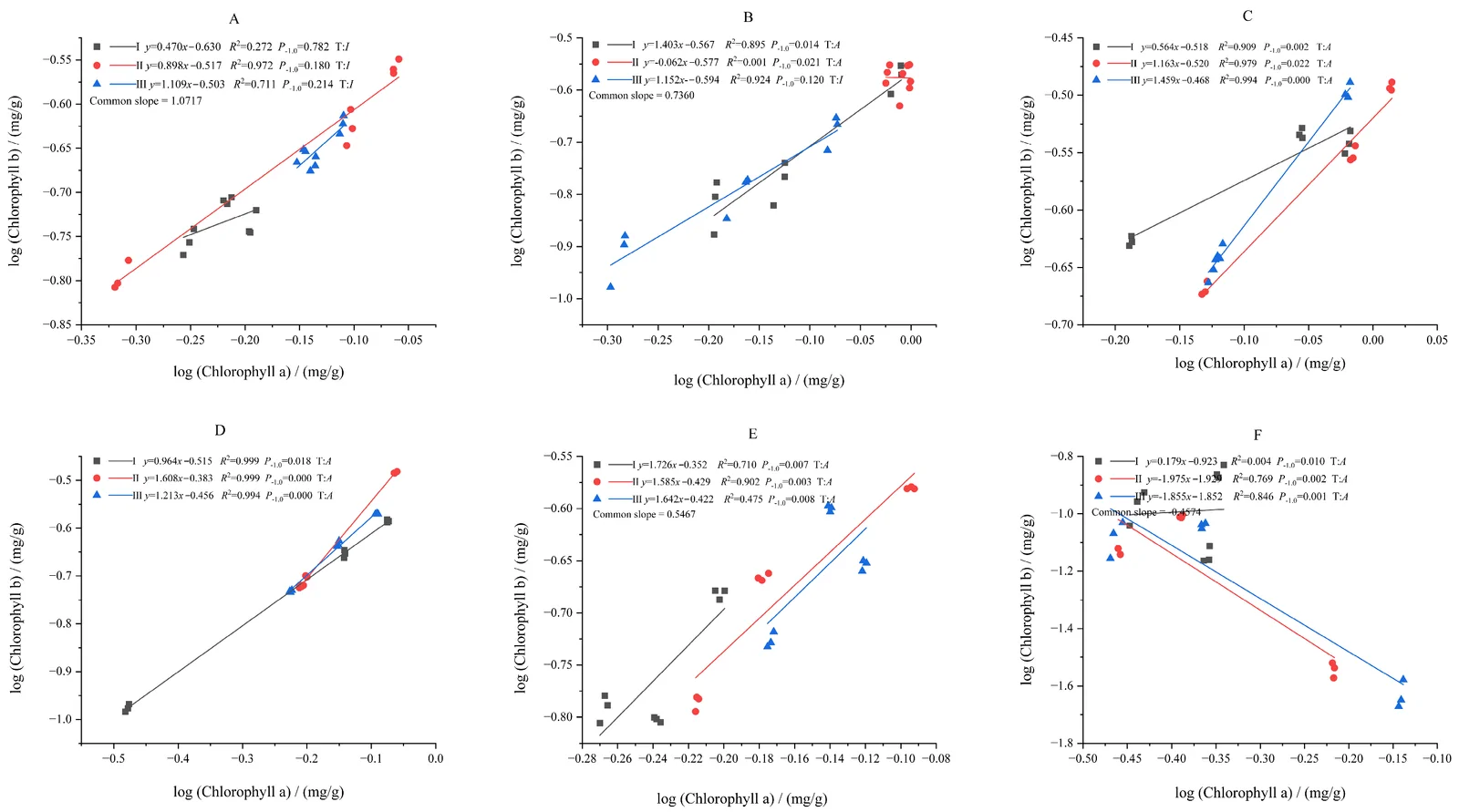
Allometric models of chlorophyll a and chlorophyll b contents. Note: Panels (**A**–**F**) correspond to seedling ages of 240 d, 330 d, 420 d, 510 d, 600 d and 690 d, respectively. Black, red and blue symbols represent Grade I, Grade II and Grade III seedlings. *P*_-1.0_ denotes the significance level for testing whether the regression slope is significantly different from the theoretical isometric value of 1.0.

With the passage of time, the allometric growth relationship between chlorophyll a and chlorophyll b generally showed a trend of isometric growth first, then allometric growth (Figure 4).

In the early seedling age (Figure 4A–C), the allometric slopes of seedlings at all grades were greater than 1, showing an allometric growth mode where the accumulation rate of chlorophyll b was faster than that of chlorophyll a. However, the fitting effects of the three grades at 240 d were generally poor (low *R*^2^).

In the middle seedling age (Figure 4D,E), the slopes were overall around or slightly greater than 1, and gradually transition to isometric growth. In the late seedling stage (Figure 4F), the slopes shift to significantly negative values, with chlorophyll b showing a decreasing trend as chlorophyll a increases, representing a negative allometric growth pattern in which the accumulation rate of chlorophyll a was significantly faster than that of chlorophyll b. At 330 d, the goodness of fit for Grades I and III improves, but the slope for Grade I remained significantly greater than 1, indicating allometric growth; whereas the slope for Grade II showed no significant difference from 1, indicating isometric growth. At 420 d, the goodness of fit for all grades was very high, and all exhibit allometric growth. At 510 d, the goodness of fit reached its peak (*R*^2^ close to 1).

### 3.5. Allometric Relationship Between Chlorophyll a and Total Chlorophyll Content in P. yunnanensis Seedlings at Different Age Grades

Regarding the relationship between chlorophyll a and total chlorophyll content (Supplementary Figure S1), as the seedling age increased from 240 d to 690 d, the relationship at 240 d and 330 d (Figure S1A,B) approached isometric growth. This stage represents a period of rapid chlorophyll construction, during which the accumulation rates of total chlorophyll and chlorophyll a are highly synchronized, with the allometric slope close to 1. This indicates that the synthesis of chlorophyll b remains balanced with that of chlorophyll a. From 420 to 600 d (Figure S1C–E), the allometric differentiation intensified, entering a phase of steady-state optimization. The slopes were generally greater than 1, indicating an allometric growth pattern in which the accumulation rate of total chlorophyll was faster than that of chlorophyll a. This suggests that the synthesis rate of chlorophyll b began to increase. Moreover, the model fit was extremely high, indicating that the accumulation relationship between the two was stable and strongly synchronized. At 690 d (Figure S1F), the slopes generally decreased. The *R*^2^ values for all grades at 690 d were generally low, and the slopes for seedlings of all grades were less than 1, indicating an allometric growth pattern in which the accumulation rate of total chlorophyll was slower than that of chlorophyll a. This does not signify a decline in chlorophyll synthesis but rather an active adjustment in resource allocation by the photosynthetic system. As seedling age increases, the light adaptation capacity of the plants strengthens, leading to a prioritization of chlorophyll a synthesis to enhance the light energy conversion efficiency of the reaction centers, while the synthesis rate of chlorophyll b slows down relatively.

### 3.6. Allometric Relationship Between Chlorophyll a and Carotenoids Content in P. yunnanensis Seedlings at Different Age Grades

A significant allometric relationship exists between chlorophyll a and carotenoids content in *P. yunnanensis* seedlings (Figure S2); however, this relationship was significantly influenced by seedling grade and age. The vast majority of regression models exhibited high coefficients of determination, particularly for Grade I, where *R*^2^ values were generally close to or equal to 1. At 240 d and 420 d, Grade I demonstrated a higher carotenoids accumulation rate than high-quality seedlings. At 510 d, the seedlings of all three grades showed similarities, with their regression lines nearly overlapping and their slope and *R*^2^ values being very close to one another. This indicates that by 510 d of growth, regardless of initial grade, the photosynthetic pigment accumulation strategies of the seedlings tend to converge, possibly entering a relatively stable period of rapid growth. At 600 d (Figure S2E) and 690 d (Figure S2F), the accumulation rate of chlorophyll a gradually increased.

### 3.7. Allometric Relationship Between Chlorophyll b and Total Chlorophyll Content in P. yunnanensis Seedlings at Different Age Grades

Regarding the relationship between chlorophyll b and total chlorophyll (Figure S3), at 240 d (Figure S3A), all three groups exhibited a nearly isometric growth trend, with a common slope of 0.9554, indicating that the accumulation of total chlorophyll and chlorophyll b was essentially synchronized; however, the fit for Grade I was low. At 330 d (Figure S3B), the fit for Grade I increased significantly, with a slope significantly less than 1, marking the formation of a stable negative allometric growth pattern: the relative accumulation rate of chlorophyll b was faster than that of total chlorophyll, indicating that Grade I seedlings prioritized resource allocation for chlorophyll b synthesis at this stage. Grade II showed a high goodness of fit but a non-significant slope, exhibiting only a weak negative allometric trend; the fit for Grade III was poor. At 420 d (Figure S3C), the slope for Grade I was significantly greater than 1, indicating a strong positive allometric growth pattern: the accumulation rate of total chlorophyll was significantly faster than that of chlorophyll b, suggesting that photosynthetic efficiency entered a period of rapid improvement. Grades II and III still maintained a negative allometric pattern. At 510 d (Figure S3D), *P. yunnanensis* seedlings entered a stable state of isometric growth, with the accumulation rates of total chlorophyll and chlorophyll b tending to become synchronized. Upon reaching 600 d and 690 d (Figure S3E,F), the negative allometric pattern between chlorophyll b and total chlorophyll intensified again, indicating an active adjustment of accumulation strategies.

### 3.8. Allometric Relationship Between Chlorophyll b and Carotenoids Content in P. yunnanensis Seedlings at Different AGE Grades

Regarding the relationship between chlorophyll b and carotenoids content (Figure S4), at 240 d (Figure S4A), the allometric relationship between carotenoids and chlorophyll b for Grade I was not significantly fitted, indicating that resource allocation in the early stage was more biased toward chlorophyll synthesis. At 330 d (Figure S4B), the allometric relationships for seedlings of all grades reached significant levels, and the pigment allocation pattern tended to stabilize. The carotenoids accumulation rate of Grade I was significantly higher than that of Grade II and III. At 510 d (Figure S4D), the slope for Grade I increased sharply, approaching isometric growth, with carotenoids and chlorophyll b accumulation being almost synchronized; in contrast, the slopes for Grade II and III were significantly lower than that of Grade I, indicating a notable lag in carotenoids accumulation rate. At 600 d (Figure S4E), the significance of the allometric relationship decreased for Grade I and II, with only Grade III maintaining a significant positive correlation. At this stage, the resource-allocation strategy of Grade I adjusted, with the accumulation rate of chlorophyll b again exceeding that of carotenoids; Grade III exhibited a lower slope, with carotenoids accumulation severely lagging, indicating insufficient photoprotective capacity. At 690 d (Figure S4F), the allometric growth pattern underwent a significant shift. Grade I still maintained a positive correlation, but the carotenoids accumulation rate lagged substantially behind that of chlorophyll b; meanwhile, the slopes for Grade II and III turned negative, indicating that as chlorophyll b accumulation increased, carotenoids content decreased instead.

### 3.9. Allometric Relationship Between Total Chlorophyll and Carotenoids Content in P. yunnanensis Seedlings at Different Age Grades

**Figure 5:**
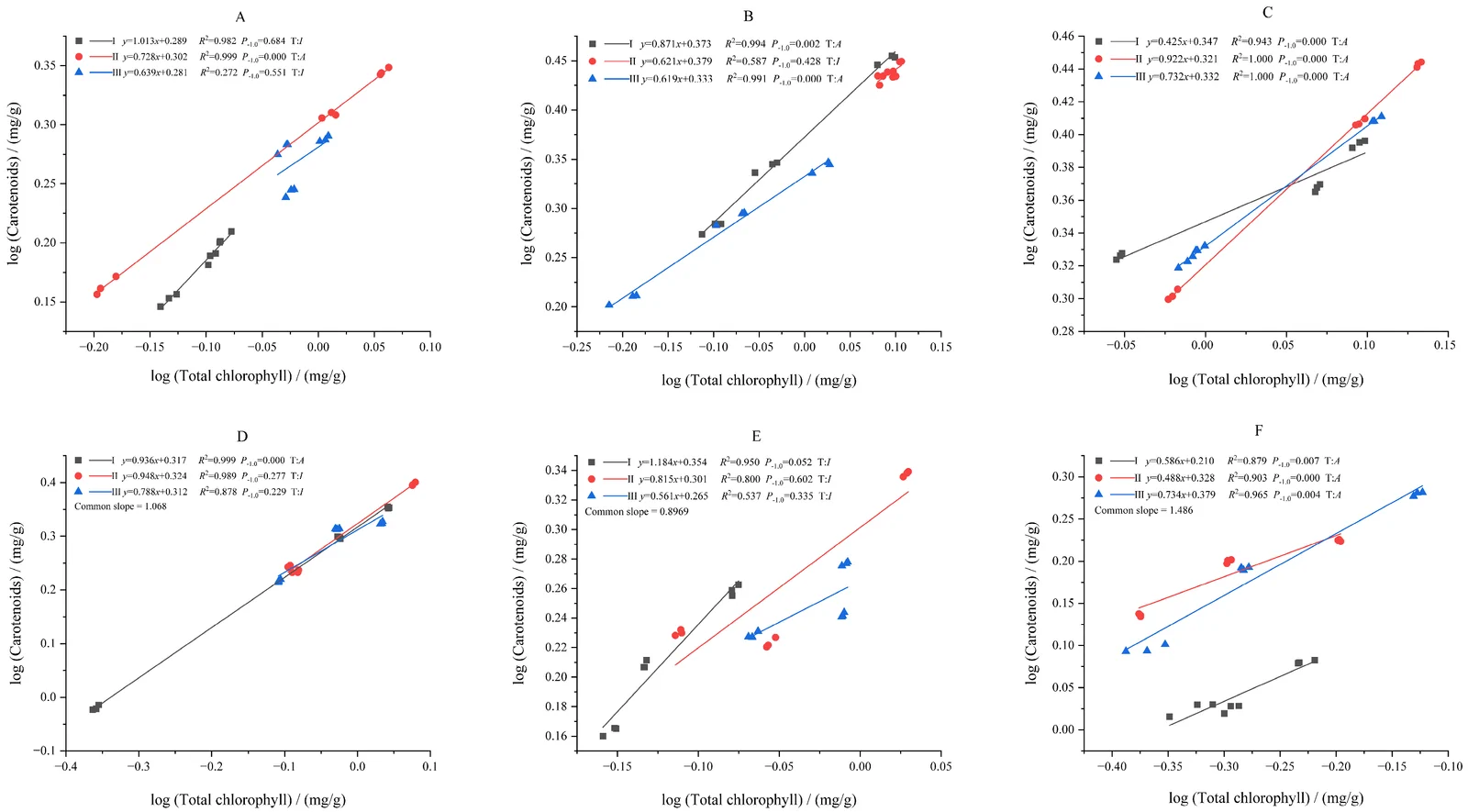
Allometric models of total chlorophyll and carotenoids contents. Note: (**A**) Allometric relationship between total chlorophyll and carotenoids contents for seedlings at 240 d; (**B**) Seedlings at 330 d; (**C**) Seedlings at 420 d; (**D**) Seedlings at 510 d; (**E**) Seedlings at 600 d; (**F**) Seedlings at 690 d. Black, red and blue symbols denote Grade I, Grade II and Grade III seedlings, respectively. *P*_-1.0_ denotes the significance level for testing whether the regression slope is significantly different from the theoretical isometric value of 1.0.

Regarding the relationship between total chlorophyll and carotenoids content (Figure 5), at 240 d (Figure 5A), although the fit for Grade I was high, the allometric relationship was not significant. At 330 d (Figure 5B), the allometric relationships for Grade I and III reached significant levels, and the carotenoids accumulation rate of Grade I was significantly higher than that of other grades. At 420 d (Figure 5C), the allometric relationships for seedlings of all grades reached extremely significant levels with very high goodness of fit, indicating that the coordinated pigment allocation pattern had become highly stable at this stage. At 510 d (Figure 5D), only the allometric relationship for Grade I remained significant, approaching isometric growth, with carotenoids and total chlorophyll accumulation being almost synchronized. At 600 d (Figure 5E), the allometric relationship for Grade I was more significant than that of other grades, with the carotenoids accumulation rate faster than that of total chlorophyll, indicating that resource allocation shifted toward the photoprotective system. At 690 d (Figure 5F), the allometric relationships for seedlings of all grades returned to significant levels, but the coordination level of other grades remained significantly lower than that of Grade I.

### 3.10. Correlation Analysis of Photosynthetic Pigments and Related Indicators in P. yunnanensis Seedlings

**Figure 6:**
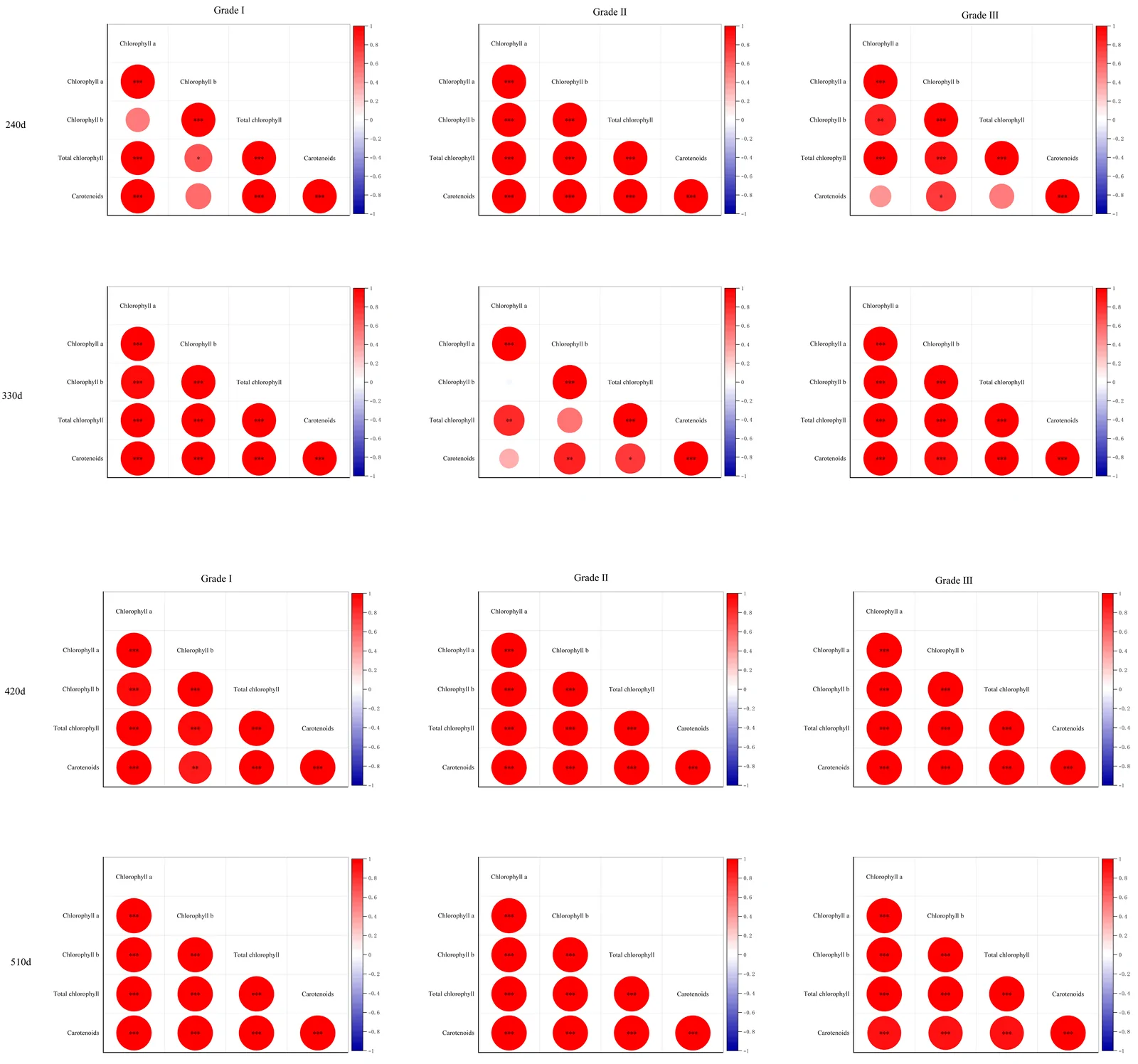

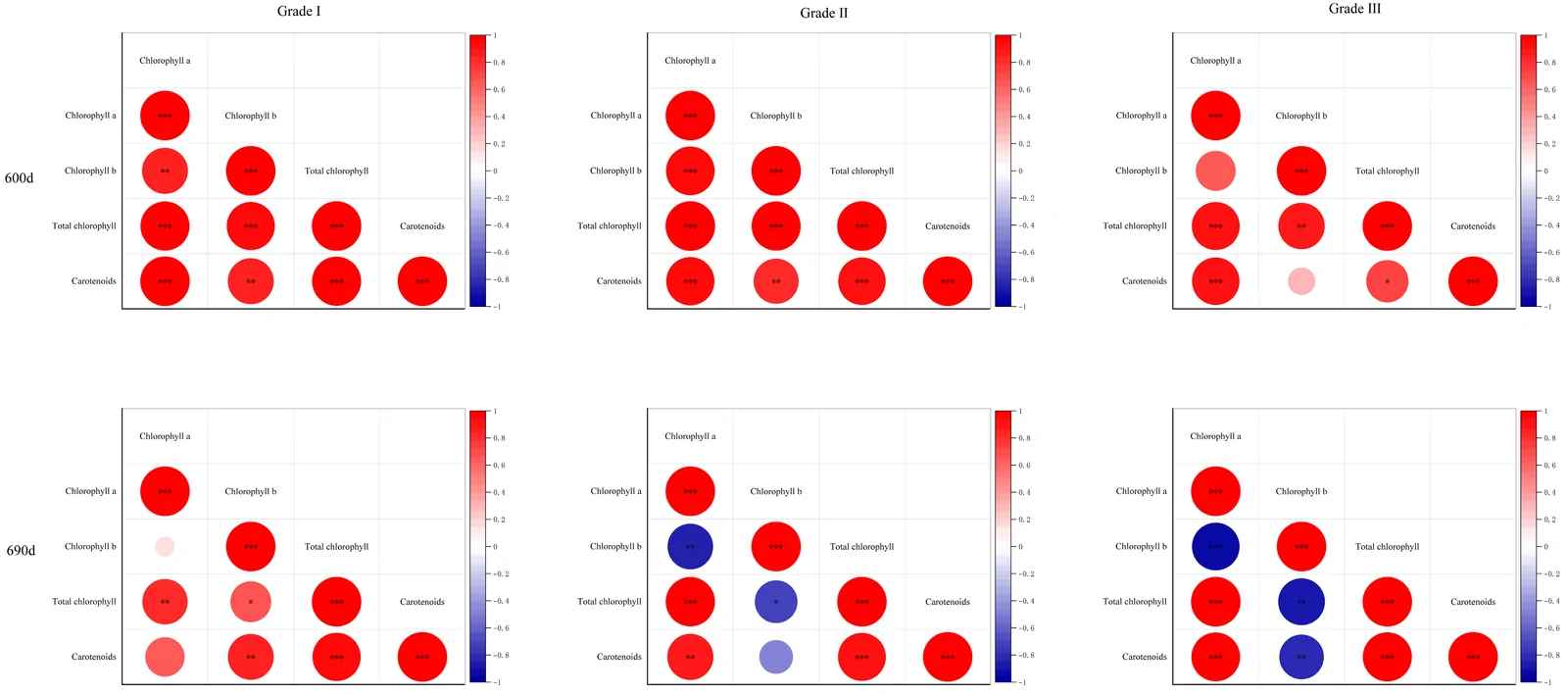
Correlation heat map of photosynthetic pigments and related indicators in *P. yunnanensis* seedlings. Each row of heatmaps corresponds to seedling ages (240 d, 330 d, 420 d, 510 d, 600 d, 690 d); each column represents Grade I, Grade II and Grade III seedlings, respectively. Circle size and color represent the magnitude and direction of Pearson correlation coefficients. Note: * *p* < 0.05, ** *p* < 0.01, *** *p* < 0.001.

In this study, differences in photosynthetic pigment content of *P. yunnanensis* seedlings influenced the early biomass of seedlings to varying degrees. Overall, the three photosynthetic pigments showed pairwise extremely significant correlations (*p* < 0.001), particularly at 420 d and 510 d (Figure 6), further reflecting the synergy of photosynthetic pigment biosynthesis. When integrating all time periods, seedling age, grade, and photosynthetic pigment content of *P. yunnanensis* seedlings showed highly significant positive correlations. From 240 d to 600 d (Figure 6), chlorophyll a, chlorophyll b, total chlorophyll, and carotenoids generally exhibited strong positive correlations across all grades, indicating a high degree of synergy in the pigment synthesis metabolic pathways, with the influence of seedling grade on the correlations gradually diminishing. At 690 d (Figure 6), the correlations showed significant differentiation. The correlation coefficients of chlorophyll b with other pigments decreased substantially, and weak negative correlations appeared in some grades. Meanwhile, carotenoids also exhibited weakened correlations with other pigments at 240 d and 330 d. This phenomenon was commonly observed in Grade II and III.

## 4. Discussion

### 4.1. Differential Analysis of Photosynthetic Pigment Content in P. yunnanensis Seedlings Across Ages and Grades

The difference between the chlorophyll a to chlorophyll b ratio reflects differences in photosynthetic apparatus structure. The high chlorophyll a/chlorophyll b ratio in Grade I may suggest a hypothetical tendency toward higher PSII reaction-center density, enhanced light-use potential and improved capacity against photodamage. Meanwhile, the unimodal pattern of the chlorophyll a to carotenoids ratio with seedling age was highly consistent with the physiological development characteristics of *P. yunnanensis* seedlings. The peak ratio at 330 d corresponds to the vigorous growth stage of the seedlings, during which chlorophyll a synthesis accelerates to meet the demands of photosynthetic carbon assimilation, hence the high chlorophyll a to carotenoids ratio; at 510 d, the seedlings were in a stable state. After 600 d, the carotenoids content increased to dissipate excess light energy and scavenge free radicals, leading to a decrease in the chlorophyll a to carotenoids ratio. The chlorophyll b to carotenoids ratio of *P. yunnanensis* seedlings exhibited significant seasonal fluctuations. At 330 d (early winter), under the stress of low temperature and photo-oxidation, Grade I actively enhanced their photoprotective adaptation strategy. From 420 to 510 d, as light and temperature conditions improved, the ratio showed compensatory recovery, and differences among grades disappeared. High-quality seedlings had a stronger capacity for photosynthetic system recovery, and the resource allocation between photoprotection and light capture rebalanced. These results indicate that high-quality seedlings exhibit stronger phenological stress adaptation, and their active photoprotective regulation at key seasonal nodes is an important physiological basis for maintaining growth advantage. The chlorophyll b to total chlorophyll ratio and the total chlorophyll to carotenoids ratio reflect the dynamic development of the photosynthetic apparatus and the adjustment of light adaptation strategies in *P. yunnanensis* seedlings, which are highly consistent with their physiological development characteristics. Similar dynamic changes in chlorophyll metabolism during seedling development have also been reported for *Pinus yunnanensis* seedlings in a recent study [32]. The gradual shifts in chlorophyll and carotenoid concentrations observed across successive sampling ages in this experiment are consistent with ontogenetic-driven pigment remodeling patterns reported for other pine seedling systems [33]. The absolute peak of total chlorophyll to carotenoids ratio at 600 d corresponds to the vigorous growth stage of established seedlings, during which total chlorophyll synthesis accelerates and photosynthetic carbon assimilation capacity reaches its peak. These results indicate that Grade I possess a stronger ability to adapt to phenological stress, and their active photoprotective regulation at key seasonal nodes (510 d) is an important physiological basis for maintaining growth advantage.

### 4.2. Ratios and Phenotypic Plasticity of Photosynthetic Pigments in P. yunnanensis Seedlings Across Ages and Grades

The high chlorophyll a to chlorophyll b ratio in Grade I indicates a lower proportion of LHCII [34], a higher density of PSII reaction centers, and greater photosynthetic efficiency and photoinhibition resistance [35]. This suggests that they are better adapted to the high-light environment of natural *P. yunnanensis* forests, and these pigment characteristics could potentially imply better out-planting performance, yet actual afforestation survival remains untested in the present study and needs field validation. The peak chlorophyll a to carotenoids ratio at 330 d corresponds to the vigorous growth stage of the seedlings, during which chlorophyll a synthesis accelerates to meet the demands of photosynthetic carbon assimilation, hence the high chlorophyll a to carotenoids ratio; after 600 d, the ratio continuously decreased, attributed to the increased photoprotective demand of older seedlings. Grade I had a higher chlorophyll a to carotenoids ratio at most seedling ages, exhibiting stronger photosynthetic capacity and growth potential, which perfectly aligns with the definition of high-quality seedlings; in contrast, Grade III had a lower chlorophyll a to carotenoids ratio, indicating weaker photosynthetic activity and higher stress sensitivity. The mid-term advantage of Grade II at 510 d reflects the dynamic adjustment of photosynthetic strategies among different grades of seedlings during growth, which is of great significance for the precision management of *P. yunnanensis* seedling cultivation. The bimodal pattern of the chlorophyll b to total chlorophyll ratio with seedling age reflects the dynamic development of the photosynthetic apparatus and the adjustment of light adaptation strategies in *P. yunnanensis* seedlings. The first peak at 240 d corresponds to the rapid growth stage of the seedlings, during which they enhance low-light capture capacity by increasing the proportion of light-harvesting antennae to meet the photosynthetic demands of rapid growth; the second peak at 600 d corresponds to the low-light stage of entering winter, during which the seedlings adapt to the nursery environment by adjusting the photosynthetic structure to improve photosynthetic efficiency under low light. The chlorophyll b to carotenoids ratio fluctuates seasonally, reflecting the periodic adaptation of the photosynthetic system to light and temperature conditions. Grade I high-quality seedlings exhibited a significantly lower ratio during both winter stress periods (330 d, 690 d), reducing low-temperature photoinhibition damage. The total chlorophyll to carotenoids ratio follows the same bimodal pattern: total chlorophyll synthesis accelerates, and photosynthetic carbon assimilation capacity reaches its peak; therefore, the total chlorophyll to carotenoids ratio is high during the two stages with suitable temperature and light conditions (240 d and 600 d). The difference in the chlorophyll a to chlorophyll b ratio is essentially a difference in the structure of the photosynthetic apparatus.

### 4.3. Variation Characteristics of Photosynthetic Pigments in P. yunnanensis Seedlings Across Ages and Grades

Chlorophyll b was the most variable component. As an auxiliary light-harvesting pigment, chlorophyll b was more sensitive to differences in individual growth, seedling grade, and environmental fluctuations, and tended to exhibit pronounced variation over the course of development; in contrast, carotenoids showed relatively low overall variability, acting as photoprotective substances for scavenging excess free radicals [36]. The variation in photosynthetic pigments of *P. yunnanensis* seedlings was mainly co-regulated by seedling age and individual grade. This indicates that Grade I have stronger photosynthetic pigment plasticity, with the magnitude of variation generally lower than that of other grades. It is worth noting that elevated variability across all seedling grades occurred at 510 d, which may represent a critical developmental transition for pigment physiology. Nevertheless, Grade I maintained lower CV values relative to Grade II and Grade III, reflecting stronger physiological stability. However, Grade I exhibited a lower coefficient of variation at all seedling ages, especially in chlorophyll a and total chlorophyll content, indicating higher consistency and adaptability in their photosynthetic physiology. In contrast, Grade III showed a higher coefficient of variation across all pigment indicators, especially for carotenoids and chlorophyll b, indicating unstable physiological status and significant individual differentiation.

### 4.4. Allometric Relationships Among Photosynthetic Pigments in P. yunnanensis Seedlings Across Ages and Grades

Photosynthesis is the foundation of all physiological activities in plants, and plant photosynthesis is highly sensitive to external environmental factors [37]. At the same time, the allometric growth index is influenced by multiple interacting factors, including the plant’s own growth status and environmental conditions [38]. The allometric relationship between chlorophyll a content and chlorophyll b content in *P. yunnanensis* seedlings changed over time [15]; chlorophyll a and chlorophyll b, as the dominant photosynthetic pigment components, can effectively indicate plant photosynthetic capacity [39]. At 240 d, the goodness of fit for chlorophyll a and chlorophyll b in each grade was very low. At this time, the seedlings may have been in the early growth stage, with the physiological system not yet stabilized, and chlorophyll synthesis was greatly affected by stochastic factors. At 330 d, Grade I exhibited an allometric growth pattern, indicating that upon entering winter, seedlings of different grades had already begun to show physiological differentiation. Grade I took the lead in adjusting in preparation for rapid growth. When the seedlings first entered the early growth stage (240 d), the proportion of chlorophyll synthesis was coordinated, with no significant differentiation among grades, and the physiological status was relatively consistent. In summer (420–510 d), with sufficient light, Grades II and III continued to increase the proportion of chlorophyll a, strengthening the reaction center to utilize strong light; the proportion of chlorophyll a in Grade I remained almost unchanged, likely having reached an optimal ratio, indicating more stable physiological regulation. All *R*^2^ values were close to 1, indicating an extremely strong relationship. During winter dormancy (600–690 d), chlorophyll began to degrade. The proportion of chlorophyll a in Grades II and III decreased significantly, which may mean faster degradation of chlorophyll a or greater retention of chlorophyll b to adapt to low light or low temperature; Grade I maintained isometric growth between chlorophyll a and total chlorophyll with a stable ratio, indicating strong physiological buffering capacity. High-quality Grade I seedlings exhibited stronger recovery capacity of the photosynthetic apparatus under fluctuating environments, which is closely associated with efficient PSII repair cycles within chloroplasts [40]. The differences in allometric patterns among different grades of *P. yunnanensis* seedlings reflect their differentiation in resource-allocation strategies, and this trade-off ultimately affects the growth rate and competitive ability of the seedlings. This is a survival strategy adopted by plants to adapt to different environmental conditions, maintaining their growth and development processes [41], to better adapt to specific habitats, demonstrating flexible growth strategies [42]. For *P. yunnanensis* seedlings, the allometric relationship between chlorophyll a and chlorophyll b was predominantly allometric, indicating that their photosynthetic organs possess strong structural plasticity. The coordinated accumulation of chlorophyll and carotenoids enhances light capture capacity [43]. This allows them to optimize the balance between light capture and light energy conversion across different seasons and light conditions, thereby maintaining high photosynthetic efficiency. In summary, Grade I exhibit resource-allocation strategies with higher adaptability to different environments, possessing high photosynthetic potential and high plasticity.

### 4.5. Integrated Correlation Analysis of Photosynthetic Pigment Indicators in P. yunnanensis Seedlings

This correlation analysis showed that seedling age, seedling grade, and various photosynthetic pigment indicators were highly significantly and positively correlated, indicating that the growth process and seedling quality are the core factors driving the synergistic construction of the photosynthetic physiological system. During the early growth period (240–600 d), chlorophyll a, chlorophyll b, total chlorophyll, and carotenoids showed extremely strong positive correlations in seedlings of all grades, indicating that the biosynthetic pathways of photosynthetic pigments were highly synergistic at this stage, with the light-harvesting system, reaction center, and photoprotective mechanism being established synchronously, and pigment accumulation following a stable proportional relationship [44]. During the late growth period (690 d), the correlations among pigments showed significant differentiation. The correlation coefficients of chlorophyll b with other pigments decreased substantially, with some grades showing weak negative correlations; the synergy between carotenoids and chlorophyll components also decreased significantly. Furthermore, the high synergy in the early growth period ensures the rapid establishment of the photosynthetic system; the stable allometric growth in the middle period enables efficient resource allocation; and the functional differentiation in the late period improves photosynthetic efficiency and stress resistance. Meanwhile, Grade I maintained higher correlation robustness, lower physiological fluctuation, and more orderly allometric transitions throughout the entire growth cycle, making them superior to Grade II and III in terms of pigment dynamics, resource allocation, and environmental adaptation. Traditional seedling-grading protocols for forest restoration mainly rely on morphological attributes such as seedling height and root-collar diameter [24]. This study provides complementary physiological evidence by comparing multi-stage photosynthetic pigment coordination among artificially graded *Pinus yunnanensis* seedlings. The present findings highlight that photosynthetic pigment traits can serve as auxiliary indicators to evaluate seedling physiological stability, which expands the existing understanding of seedling-quality assessment for subtropical native conifers.

## 5. Conclusions

This study explored photosynthetic pigment traits, trait variability and allometric resource-allocation patterns across ontogenetic stages for height-graded *Pinus yunnanensis* seedlings. The findings demonstrate that seedling age and seedling grade jointly shape photosynthetic pigment metabolism, stoichiometry, phenotypic variation and allometric scaling relationships.

Grade I seedlings displayed superior physiological performance characterized by stable pigment composition, low phenotypic variation and coordinated pigment accumulation. Physiological transitions occurred during seedling ontogeny, and Grade I maintained well-balanced trade-offs between photosynthetic carbon assimilation and photoprotection, whereas Grade II and Grade III exhibited stronger pigment-related phenotypic fluctuation. These observations suggest that stable and synergistic regulation of the photosynthetic pigment system constitutes an important physiological basis for the better performance of high-grade seedlings.

Findings from this work provide physiological evidence for physiological divergence among height-graded seedlings, and offer potential reference indicators for the early physiological evaluation of *Pinus yunnanensis* seedlings. In addition, seedling grading was implemented according to seedling height at 240 d; post-grading physiological differences may partly stem from pre-existing individual divergence prior to grading. Nevertheless, all observations are derived from greenhouse common-garden cultivation, and caution should be exercised when extrapolating these physiological patterns to complex field afforestation environments. Further verification is needed to determine whether pigment-related physiological differences persist following out-planting. Integration of multi-trait indicators is recommended to refine seedling-quality evaluation systems for native conifer species in Southwest China.

## Data Availability

The original contributions presented in this study are included in the article/Supplementary Materials. Further inquiries can be directed to the corresponding author.

## Supplementary Materials

The following supporting information can be downloaded at: https://www.mdpi.com/article/10.3390/biology15201844/s1, Figure S1. Allometric models of chlorophyll a and total chlorophyll contents; Figure S2. Allometric models of chlorophyll a and carotenoids contents. Figure S3. Allometric models of chlorophyll b and total chlorophyll contents. Figure S4. Allometric models of chlorophyll b and carotenoids contents. Table S1: Descriptive statistics and classification of seedlings based on initial seedling height.

## References

[1] Tang G.P., Liao J.Z., Xu Y.L., Cai N.H. (2025). Effects of Top-Pruning Intensity Gradient on Root System Architecture and Allometric Patterns in *Pinus yunnanensis* Franch. Seedlings. Plants.

[2] Zhou F.L., Shi F.Q. (2005). Selection on Superior Families of *Betula alnoides* Hamilt. J. Yunnan Agric. Univ..

[3] da Silva R.B.G., Simões D., Wendling I., do Prado D.Z., Sartori M.M.P., Bertholdi A.A.d.S., da Silva M.R. (2023). Leaf Angle as a Criterion for Optimizing Irrigation in Forest Nurseries: Impacts on Physiological Seedling Quality and Performance after Planting in Pots. Forests.

[4] Kamanga R.M., Stevens J.C., Tuwadala K., Kachitsa S.L., Nyasulu G., Mwale V.M. (2026). Morpho-physiological seedling quality assessment of selected ornamental plant species produced by nurseries in Lilongwe, Malawi. Discov. Agric..

[5] Grossnickle S.C., MacDonald J.E. (2018). Seedling Quality: History, Application, and Plant Attributes. Forests.

[6] Wang Q.B., Yan T.Y., Wang Y., Li Y.Q., Wang D., Chen S., Chen L., Tang J.R., Cai N.H., Xu Y.L. (2022). Classification and allometric growth of one-year-old *Pinus yunnanensis* seedlings from different provenances. J. Cent. South Univ. For. Technol..

[7] Kim I., Kwak M., Kim J., Woo S. (2025). Short-term physiological responses of Pinus koraiensis Siebold & Zucc. and Chamaecyparis obtusa (Siebold & Zucc.) Endl. seedlings to elevated CO_2_. Front. For. Glob. Change.

[8] Wang D., Li X.Y., Yan T.Y., Chen S., Xu Y.L., Cai N.H., Xu D.B. (2023). Effects of stubble cutting in different seasons on biomass allocation and allometric growth of *Pinus yunnanensis*. Southwest China J. Agric. Sci..

[9] Normand F., Bissery C., Damour G., Lauri P.E. (2008). Hydraulic and mechanical stem properties affect leaf-stem allometry in mango cultivars. New Phytol..

[10] Huang J., Wang D.X., Yang S., Zheng C.F., Lu Z.Y., Xu Y.L. (2023). Effect on chlorophyll content and allometric growth relationship of *Pinus yunnanensis* seedlings by foliar spraying of nitrogen and phosphorus. J. Southwest For. Univ. (Nat. Sci.).

[11] Murphy B.K., Lorusso S., Isabel N., Ensminger I. (2025). Contrasting effects of warming and drought on autumn phenology of photosynthesis and growth in white spruce. Plant Physiol..

[12] Croce R., van Amerongen H. (2014). Natural strategies for photosynthetic light harvesting. Nat. Chem. Biol..

[13] Kong D., Xu Y.L. (2025). Effects of foliar nitrogen and phosphorus fertilization on growth, biomass distribution of *Pinus yunnanensis*. J. Cent. South Univ. For. Technol..

[14] Wang D., Li J.F., Li Y.Q., Yan T.Y., Chen S., Xu Y.L., Cai N.H. (2022). Allometric growth and phenotypic plasticity of *Pinus yunnanensis* at different seedling ages. J. Cent. South Univ. For. Technol..

[15] Poorter H., Nagel O.W. (2000). The role of biomass allocation in the growth response of plants to different levels of light, CO_2_, nutrients and water: A quantitative review. Aust. J. Plant Physiol..

[16] West G.B., Brown J.H., Enquist B.J. (1997). A General Model for the Origin of Allometric Scaling Laws in Biology. Science.

[17] Huang J.Y., Yu H.L. (2016). Review on the mechanism models of allometric scaling. J. Plant Ecol..

[18] Niklas K.J. (2004). Modelling Below- and Above-ground Biomass for Non-woody and Woody Plants. Ann. Bot..

[19] Mokany K., Raison R.J., Prokushkin A.S. (2006). Critical analysis of root:shoot ratios in terrestrial biomes. Glob. Change Biol..

[20] Jackson R.B., Schenk H.J., Jobbágy E.G., Canadell J., Colello G.D., Dickinson R.E., Field C.B., Friedlingstein P., Heimann M., Hibbard K. (2000). Belowground consequences of vegetation change and their treatment in models. Ecol. Appl..

[21] Neuwirthová E., Stejskal J., Lhotáková Z., Korecký J., Čepl J., Nikodem A., Panzarová K., Albrechtová J., Lstibůrek M. (2025). Drought Response and Genetic Variation in Scots Pine Seedlings’ Provenances: Insights From High-Throughput Phenotyping for Climate-Resilient Forestry. Evol. Appl..

[22] Pélabon C., Hilde C.H., Einum S., Gamelon M. (2020). On the use of the coefficient of variation to quantify and compare trait variation. Evol. Lett..

[23] Zhu M.M., Zhu Q.G., Li B., Wang Z.Y., Pan S.H. (2022). Research progress on allometric relationships among plant modules. Anhui Agric. Sci. Bull..

[24] Haase D.L. (2008). Understanding Forest Seedling Quality: Measurements and Interpretation. Tree Plant. Notes.

[25] Martini F., Xia S.-W., Zou C., Goodale U.M. (2020). Seedling growth and survival responses to multiple soil properties in subtropical forests of south China. For. Ecol. Manag..

[26] Lu X.M., Zhou C.F., An S.Q., Fang C., Zhao H., Yang Q., Yan C. (2007). Phenotypic plasticity, allometry and invasiveness of plants. Chin. J. Ecol..

[27] Niklas K.J. (1995). Size-dependent Allometry of Tree Height, Diameter and Trunk-taper. Ann. Bot..

[28] Li Y.Q. (2021). Response of *Pinus yunnanensis* Seedlings to Different Combined Applications of Nitrogen and Phosphorus. Master’s Thesis.

[29] Han W.X., Fang J.Y. (2008). Review on the mechanism models of allometric scaling laws: 3/4 vs. 2/3 power. J. Plant Ecol..

[30] Warton D.I., Duursma R.A., Falster D.S., Taskinen S. (2012). smatr 3—An R package for estimation and inference about allometric lines. Methods Ecol. Evol..

[31] Warton D.I., Wright I.J., Falster D.S., Westoby M. (2006). Bivariate line-fitting methods for allometry. Biol. Rev. Camb. Philos. Soc..

[32] Li R.L., Wang Q.B., Li S.L., Chen L., Cai N.H., Xu Y.L. (2025). Study on the growth characteristics of *Pinus yunnanensis* seedlings based on hormonal regulation and chlorophyll metabolism. Sci. Rep..

[33] Oluborode J., Chadzinikolau T., Formela-Luboińska M., Ye Z.-P., Robakowski P. (2024). Adaptive significance of age- and light-related variation in needle structure, photochemistry, and pigments in evergreen coniferous trees. Photosynth. Res..

[34] Ruban A.V., Young A.J., Horton P. (1996). Dynamic properties of the minor chlorophyll a/b binding proteins of photosystem II, an in vitro model for photoprotective energy dissipation in the photosynthetic membrane of green plants. Biochemistry.

[35] Kim E., Lee D., Sakamoto S., Jo J.-Y., Vargas M., Ishizaki A., Minagawa J., Kim H. (2025). Network analysis with quantum dynamics clarifies why photosystem II exploits both chlorophyll a and b. Sci. Adv..

[36] Maoka T. (2020). Carotenoids as natural functional pigments. J. Nat. Med..

[37] Wang Q.B., He B., Lu Z.Y., Yang Z.X., Zheng C.F., Wang Y., Cai N.H., Xu Y.L., Jin J.H., Wang D.X. (2024). Variation of Growth Traits and Allometric Growth of Seedlings of *Pinus yunnanensis* from Different Provenances. J. Southwest For. Univ. (Nat. Sci.).

[38] Chen G.P., Zhao W.Z., He S.X., Fu X. (2016). Biomass Allocation and Allometric Relationship in Aboveground Components of Salix psammophila Branches. J. Desert Res..

[39] Wang D., Li Y.Q., Sun J.W., Li J.F., Chen S., Xu Y.L., Cai N.H. (2021). Allometric growth of *Pinus yunnanensis* seedlings from different families. Bull. Bot. Res..

[40] Järvi S., Suorsa M., Aro E.-M. (2015). Photosystem II repair in plant chloroplasts--Regulation, assisting proteins and shared components with photosystem II biogenesis. Biochim. Biophys. Acta.

[41] Chen J.L., Wu J.W., Duan G.H., Liu Y.X., Zhu W.S., Han J.C. (2026). Effects of shading on needle physiology of *Pinus yunnanensis* seedlings at different ages. J. Northeast. For. Univ..

[42] Croft H., Chen J.M., Luo X., Bartlett P., Chen B., Staebler R.M. (2017). Leaf chlorophyll content as a proxy for leaf photosynthetic capacity. Glob. Change Biol..

[43] Frank H.A., Cogdell R.J. (1996). Carotenoids in photosynthesis. Photochem. Photobiol..

[44] Björkman O., Demmig-Adams B., Schulze E.-D., Caldwell M.M. (1995). Regulation of Photosynthetic Light Energy Capture, Conversion, and Dissipation in Leaves of Higher Plants. Ecophysiology of Photosynthesis.

