# Supplementary material for: Coordination and Variation of Photosynthetic Pigments Across Seedling Ages and Grades Reveal Adaptive Strategies in Pinus yunnanensis
Source: Biology (Basel). 2026 Oct 14;15(20):1844. doi: 10.3390/biology15201844 (PMC13701417; doi:10.3390/biology15201844)
Supplement: Supplementary file 1 [file biology-15-01844-s001.zip › Supplementary_Figures_S1-S4.pdf]

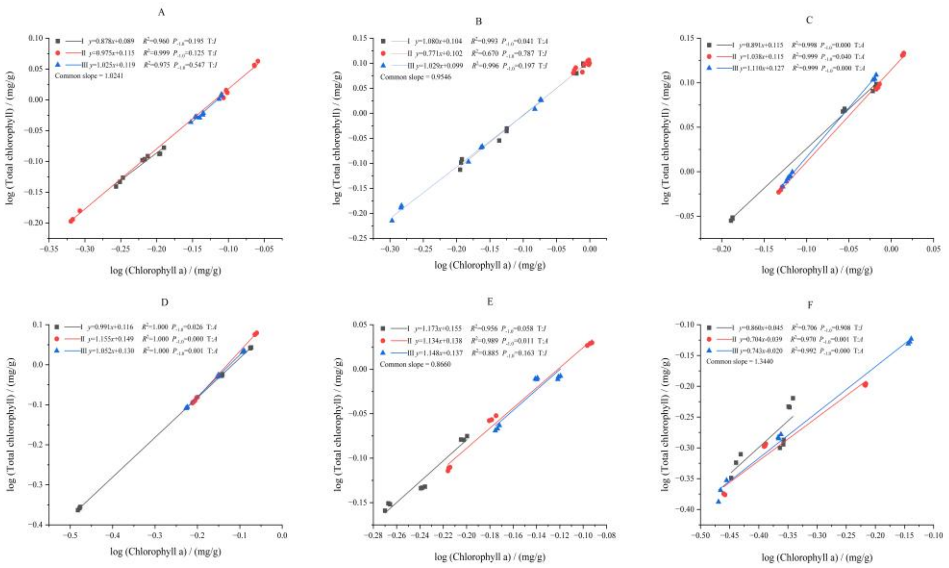

Figure S1. Allometric models of chlorophyll a and total chlorophyll contents. Different lowercase letters indicate significant differences among different seedling age treatments (*P* < 0.05)

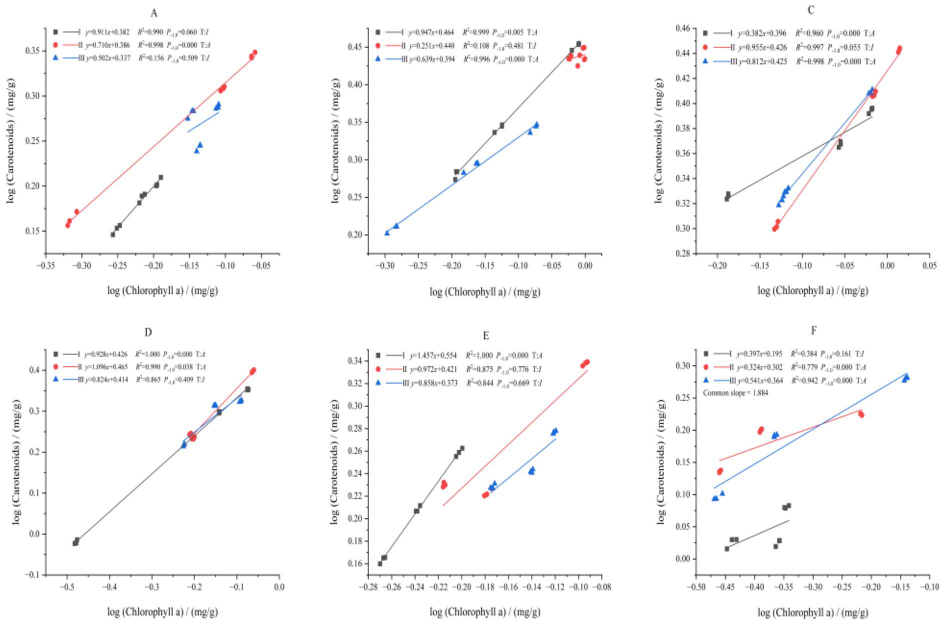

Figure S2. Allometric models of chlorophyll a and carotenoids contents. Different lowercase letters indicate significant differences among different seedling age treatments (P < 0.05)

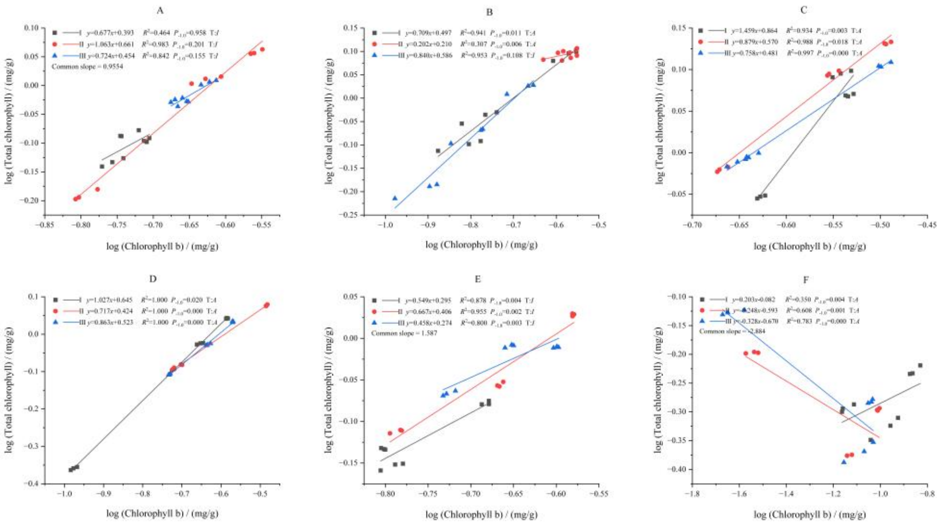

Figure S3. Allometric models of chlorophyll b and total chlorophyll contents. Different lowercase letters indicate significant differences among different seedling age treatments ($P < 0.05$)

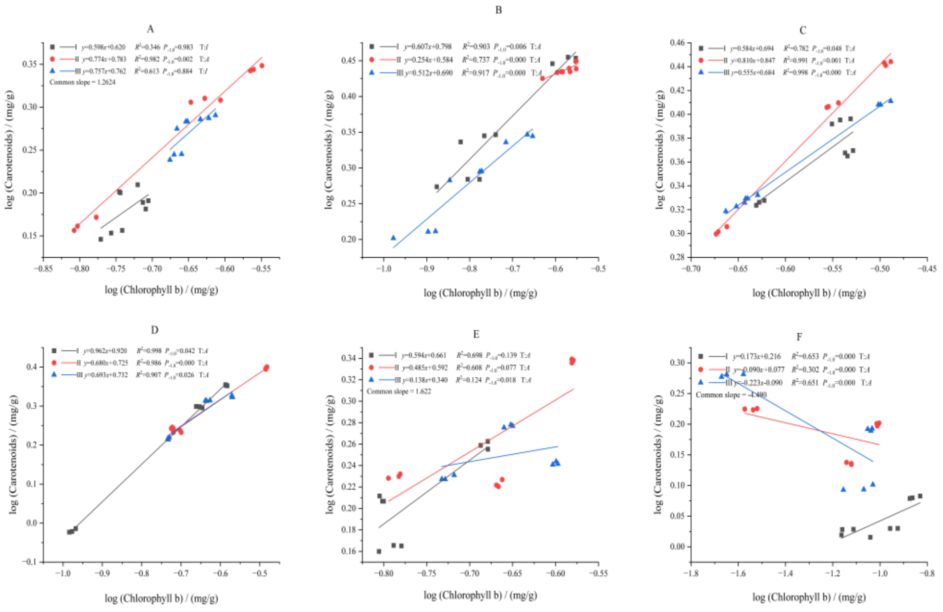

Figure S4. Allometric models of chlorophyll b and carotenoids contents. Different lowercase letters indicate significant differences among different seedling age treatments ($P < 0.05$)
